# The Land of Golden Day

**Published:** 1888-04-21

**Authors:** 


### THE LAND OF GOLDEN DAY.

A sickness like to death came o'er my child,
  I dreamed I saw him stand
Upon the darkening shore,
From whence a shadowy vessel bare him o'er
  To a wide peaceful land:
Goodly, and beauteous, and undefiled—
  The land of day,
  The Land of Golden Day!

Then, as the dusky vessel left my dream
  He stepped upon the strand,—
Treading the peaceful shore
He heard rich harpings of the harpers pour
  Over the lilied land,
Where azure waves of life do move and gleam
  In the land of day,
  The Land of Golden Day!

"Go not, oh child," I cried with voice of woe,
  "Go not to the angel band,
Come back to me once more."
But, ah! he turned him from the peaceful shore,
  Led by an angel's hand!
No more the bitter wail of pain to know
  In the land of day,
  The Land of Golden Day!

Kind Heaven! I woke—I woke to earth and love,
  I touched my baby's hand,
Unknowing joy before;
He had not passed unto the peaceful shore,
  To the calm angel band!
Life had been sent him from its wealth above
  In the land of day,
  The Land of Golden Day!

—*Lina Forster.*

